# Multisystem burden in cirrhosis: lessons from a marginalised population

**DOI:** 10.1016/j.lanwpc.2025.101645

**Published:** 2025-07-25

**Authors:** Pojsakorn Danpanichkul, Jeffrey V. Lazarus, Ju Dong Yang, Amit G. Singal

**Affiliations:** a Department of Internal Medicine, Texas Tech University Health Sciences Center, Lubbock, TX, USA; b CUNY Graduate School of Public Health and Health Policy (CUNY SPH), New York, NY, USA; c Faculty of Medicine and Health Sciences, University of Barcelona, Barcelona, Spain; d Barcelona Institute for Global Health (ISGlobal), Hospital Clínic, University of Barcelona, Barcelona, Spain; e Division of Digestive and Liver Diseases, UT Southwestern Medical Center, Dallas, TX, USA; f Karsh Division of Gastroenterology and Hepatology, Comprehensive Transplant Center, Samuel Oschin Comprehensive Cancer Institute, Cedars-Sinai Medical Center, Los Angeles, CA, USA

A new population-based study by Bhasker and colleagues^1^ offers the first detailed insight into cause-specific mortality in Australians with cirrhosis, uncovering profound disparities. The study found that although liver disease itself accounted for roughly half of all deaths in both First Nations and non-Indigenous patients with cirrhosis, First Nations patients died almost a decade younger on average and suffered significantly higher mortality from non-liver causes. Specifically, deaths from cardiovascular disease, diabetes, and infections were 60% more frequent in First Nations Australians with cirrhosis compared to non-Indigenous Australians. Notably, 10-year liver-related mortality did not differ by indigenous status, with the excess in other causes driving the mortality gap. Clinically, this signals that managing cirrhosis in First Nations patients cannot focus on the liver alone. Instead, a holistic, multidisciplinary approach to care is imperative: cardiometabolic comorbidities and infection risks, along with commercial and social determinants of health, must be addressed alongside liver disease.^2^ By understanding these patterns, the study underscores the need to broaden clinical focus beyond hepatology, a shift with immediate relevance for practitioners caring for Indigenous patients with chronic liver disease.

The implications of this study extend beyond Australia. Globally, Indigenous populations bear a disproportionate burden of chronic liver disease. In Canada, liver disease ranks among the leading contributors to the mortality gap between First Nations and non-Indigenous populations.^3^ In some communities, liver-related mortality is up to six times higher.^3^ Similarly, in the United States, American Indian and Alaska Native communities face the highest cirrhosis and digestive disease mortality rates nationwide, more than double those seen in White non-Hispanics.^4^ These patterns point to common underlying issues, social inequities and inadequate access to preventive care, that transcend national borders.

Bhasker et al.'s findings mirror this international trend: Indigenous populations with cirrhosis face excess mortality driven not only by liver disease but also by cardiovascular, metabolic, and infectious causes.^5^ These outcomes reflect a convergence of biological risk, social determinants, and systemic healthcare disparities.^6^ As such, the study underscores the need to expand cirrhosis care beyond the liver, emphasizing multidisciplinary care models—or even a medical home approach, for patients with liver disease. This is especially critical in the context of non-viral etiologies such as metabolic dysfunction and alcohol-associated liver disease, which exert wide-ranging effects on multiple organ systems.^7^

Interestingly, this study noted a lower prevalence of metabolic-dysfunction associated steatohepatitis (MASH) cirrhosis among First Nations patients, despite high diabetes rates.^1^ It is possible this paradox may reflect that competing risk factors, such as alcohol use or viral hepatitis, may overshadow MASH as the dominant cirrhosis etiology in this population.^8^ Some First Nations patients may fulfill criteria for metabolic dysfunction and alcohol-associated liver disease (MetALD), while others classified as having ALD may still have underlying metabolic risk factors. Public health strategies targeting preventable contributors, chronic viral hepatitis, metabolic syndrome, alcohol use, are crucial for upstream prevention^9^ along with mindful language choices to tackle potential stigma.^2^

Despite new insights from this study, several clinical questions remain. Granular data on care processes, such as cardiovascular risk factor control, or timeliness of infection management, would help identify where interventions could save lives. For instance, First Nations patients might be less likely to be referred for specialty care after discharge or may be less likely to attend follow-up appointments. Evidence from the United States highlights that racial and ethnic minorities with liver disease often face significant barriers to care, including medical mistrust and lower health literacy, which may contribute to disparities in outcomes.^10^ Understanding whether similar patterns exist in Australia could inform equity-focused policy changes. Future research should emphasize implementation science and the evaluation of intervention trials that test scalable, culturally appropriate models of care. The research agenda must shift from documenting disparities to identifying and validating strategies that effectively reduce preventable deaths and drive structural improvements in liver disease care for Indigenous populations.

## Contributors

Writing, original draft—Pojsakorn Danpanichkul.

Writing, review, and editing—Amit G. Singal, Jeffrey V. Lazarus, Ju Dong Yang.

All authors have read and approved the final version of the manuscript for submission.

## Declaration of interests

**Jeffrey V. Lazarus** acknowledges grants to ISGlobal from AbbVie, Boehringer Ingelheim, Echosens, Gilead Sciences, Madrigal, Moderna, MSD, Novo Nordisk, Pfizer, and Roche Diagnostics, consulting fees from Echosens, GSK, Novavax, Novo Nordisk, Pfizer, and Prosciento, and payment or honoraria for lectures from AbbVie, Echosens, Gilead Sciences, Janssen, Moderna, MSD, Novo Nordisk, and Pfizer, outside of this work. **Amit G. Singal** has served as a consultant or on advisory boards for Genentech, AstraZeneca, Eisai, Exelixis, Bayer, Elevar, Boston Scientific, Sirtex, FujiFilm Medical Sciences, Exact Sciences, Helio Genomics, Roche, Abbott, Glycotest, Curve Bio, ImCare, and GRAIL. **Ju Dong Yang** consults for AstraZeneca, Eisai, Exact Sciences, and FujiFilm Medical Sciences. **Pojsakorn Danpanichkul** denied conflict of interest.
